# Anti-microbial activities of pomegranate rind extracts: enhancement by cupric sulphate against clinical isolates of *S. aureus*, MRSA and PVL positive CA-MSSA

**DOI:** 10.1186/1472-6882-9-23

**Published:** 2009-07-27

**Authors:** Simon WJ Gould, Mark D Fielder, Alison F Kelly, Declan P Naughton

**Affiliations:** 1 School of Life Sciences, Kingston University, Kingston upon Thames, London KT1 2EE, UK

## Abstract

**Background:**

Recently, natural products have been evaluated as sources of antimicrobial agents with efficacies against a variety of micro-organisms.

**Methods:**

This report describes the antimicrobial activities of pomegranate rind extract (PRE) singularly and in combination with cupric sulphate against methicillin-sensitive and -resistant *Staphylococcus aureus *(MSSA, MRSA respectively), and Panton-Valentine Leukocidin positive community acquired MSSA (PVL positive CA-MSSA).

**Results:**

PRE alone showed limited efficacy against MRSA and MSSA strains. Exposure to copper (II) ions alone for 2 hours resulted in moderate activity of between 10^2 ^to 10^3 ^log_10 _cfu mL^-1 ^reduction in growth. This was enhanced by the addition of PRE to 10^4 ^log_10 _cfu mL^-1 ^reduction in growth being observed in 80% of the isolates. However, the PVL positive CA-MSSA strains were more sensitive to copper (II) ions which exhibited moderate activities of between 10^3 ^log_10 _cfu mL^-1 ^reduction in growth for 60% of the isolates.

**Conclusion:**

PRE, in combination with Cu(II) ions, was seen to exhibit moderate antimicrobial effects against clinical isolates of MSSA, MRSA and PVL positive CA-MSSA isolates. The results of this study indicate that further investigation into the active ingredients of natural products, their mode of action and potential synergism with other antimicrobial agents is warranted. This is the first report of the efficacy of pomegranate against clinical PVL positive CA-MSSA isolates.

## Background

Considerable emphasis is being placed on combating multi-drug resistant bacteria in the clinical setting. These proposed infection control measures encompass the study of hospital cleaning measures, prevention of transmission by healthcare workers and the development of new antimicrobial agents [1-3]. During the year beginning in April 2005, methicillin resistant *Staphylococcus aureus *(MRSA) bacteraemia cases in England totalled 7,087 [4]. Methicillin sensitive *Staphylococcus aureus *(MSSA) are also resistant to multiple antibiotics [5], however these isolates respond better to treatment than MRSA counterparts. The community acquired MSSA (CA-MSSA) can produce the Panton-Valentine Leukocidin (PVL) toxin which is of particular concern as infections with these organisms have resulted in increased levels of morbidity and mortality [6].

Recently, studies have been redirected towards evaluating traditional medicines as sources of antimicrobial agents [7-10]. A wide range of natural products have been screened for activity against *S. aureus*, including the pomegranate (*Punica granatum*). In a study by Machado *et al*. [11] various extracts of pomegranates (in ethanol, chloroform, ethyl acetate, butanol and water) all exhibited activities against MSSA and MRSA. A further study by Braga *et al*. [12] demonstrated the inhibition of growth and release of enterotoxin by some *S. aureus *isolates.

Numerous metal containing-antimicrobial agents have been reported despite advanced protective mechanisms for the detoxification of heavy metal ions being found in many bacteria [13]. The enhancement of the anti-bacteriophage and antimicrobial activities exhibited by pomegranate rind extracts (PRE) by the addition of metal ions has been reported [14,15]. Stewart *et al*. reported remarkable anti-bacteriophage activities for the combination of pomegranate rind extracts with ferrous salts. However, this combination was found to exhibit activity for a short period probably owing to instability. Previous work carried out by the current authors demonstrated that PRE, in combination with different metals, showed an antibacterial effect against a range of laboratory strains of both Gram positive and negative bacteria [15]. This study showed that PRE with the addition of CuSO_4 _demonstrated inhibitory effects against three Gram negative strains of bacteria: *Pseudomonas aeruginosa *(NCTC 950), *Proteus mirabilis *(NCTC 7827) and *Escherichia coli *(NCTC 12241), with kill rates from 10^8 ^cfu mL^-1 ^to no detectable growth within 30 minutes. Against *S. aureus *this combination (PRE/CuSO_4_) reduced the bacterial population by approximately 10^2^cfu mL^-1^; however, the addition of a stabilising agent (vitamin C) to the combination resulted in no detectable growth after 30 minutes.

The aim of this study was to explore the potential role for the PRE/Cu(SO_4_) with the addition of a stabilising agent against clinical isolates of *S. aureus*. Thirty isolates were tested which included MRSA (n = 10), MSSA (n = 10) and PVL positive CA-MSSA (n = 10).

## Methods

### Preparation of pomegranate rind extract

Pomegranate rind extract (PRE) was prepared by cutting rind into small squares (approximately 5 mm^2^) which were dried at 55°C for 24 hours, and stored in an air tight container in the dark until further use. A 10 g sample of dry rind was added to 150 mL distilled water and placed in a shaker (at 80 rpm) at room temperature for 24 hours [9]. The crude extract was passed though muslin and a Whatman filter No.1 to remove the particulate matter, prior to filter sterilising by passing through a 0.2 μm filter (Millipore), into a sterile bottle. The extract was stored at -20°C for future use.

### Bacterial isolates

Clinical isolates of MRSA (n = 10), MSSA (n = 10) and PVL positive CA-MSSA (n = 10) were used in the study. The MRSA and MSSA isolates were collected from the Royal Marsden hospital (London, UK) and the PVL positive CA-MSSA isolates were collected from the Devon and Exeter hospital (UK). Identification of all isolates was determined by Gram stain and Staphylase Test (Oxoid, UK) which were conducted in house. The isolates were cultured aerobically overnight on nutrient agar (Oxoid) at 37°C and then frozen in cryovials (Pro-labs, UK) at -80°C until required. Prior to use all isolates were passaged twice on nutrient agar aerobically at 37°C. In all assays inocula were prepared by using overnight cultures on nutrient agar that were then suspended in Ringer's solution (Oxoid) to a turbidity equivalent to 0.5 McFarland (1.5 × 10^8 ^cfu mL^-1^).

### Antibiotic sensitivity testing

The three groups of bacteria were tested against a specific panel of antibiotics for that group, in house, using standard operating procedures defined by the British Society for Antimicrobial Chemotherapy [16]. All antibiotics were purchased from MAST diagnostic (UK). The MRSA and MSSA isolates were tested against cefotixitin (10 μg), penicillin (1 unit), erythromycin (5 μg), gentamicin (10 μg), rifampicin (2 μg), cancomycin (5 μg), mupirocin (5 μg) and cefuroxime (5 μg). The PVL CA-MSSA isolates were tested against penicillin (1 unit) and methicillin (5 μg).

### Antimicrobial activity of PRE with the addition of cupric sulphate

All reagents were purchased from Sigma-Aldrich Chemical Co. (Poole, Dorset) and distilled water was used as diluent throughout. The method used was an adaptation of that described by McCarrell *et al*. [15]. Briefly, overnight cultures on nutrient agar were suspended in Ringer's solution (Oxoid) to a turbidity equivalent to 0.5 McFarland (1.5 × 10^8 ^cfu mL^-1^). An aliquot of the PRE extract (330 μL) was added to 700 μL of the freshly prepared solutions (4.8 mM) of cupric sulphate (CuSO_4_,); the final solution was protected from light [15].

The appropriate bacterial dilution was prepared and 50 μL placed in a sterile Eppendorf micro-centrifuge tube (SLS, UK) with 100 μL of the extract/metal salt solution (Lambda buffer used for control). Following incubation of the sample for 2 hours at room temperature, the activity of the bactericidal agent was neutralized by adding an equal volume of 2% (v/v) Tween-80 (Sigma-Aldrich Chemical Co., UK) in Lambda buffer [15]. Serial dilutions were prepared in Ringer's solution, 10 μL of each dilution was spotted onto a nutrient agar plate and incubated aerobically for 24 hours at 37°C. Each assay was carried out in triplicate.

### Antimicrobial activity of PRE and cupric sulphate with the addition of different concentrations of vitamin C

The antimicrobial assay was carried out as previously stated McCarrell *et al*., [15] with the following modification. Before adding the cupric salt solution to PRE, vitamin C was added to the cupric salt. Varying concentrations of vitamin C were added to the copper(II) salt solution, comprising the following ratios; 1:1 (4.8 mM), 1:5 (24 mM), 1:20 (96 mM) (cupric salt: vitamin C), 700 μL of which was added to PRE.

### Minimum inhibition concentration (MIC) determination of PRE and CuSO_4_

Micro-dilution plates were prepared with freeze-dried PRE or CuSO_4_solution which was added to sterile water at a concentration of 800 mg mL^-1^. The plates were prepared as follows; 50 μL of four-times strength Iso-Sensitest broth (Oxoid, UK) was added to the first row (A) of wells and 50 μL of double strength Iso-Sensitest broth was added to all remaining wells. To the first row of wells 50 μL of the PRE was added and mixed, 50 μL of broth from row A was transferred to row B and mixed, this process was continued to row F. Finally, 50 μL of broth was removed from well F and discarded. The overnight cultures were suspended in Ringer's solution to a turbidity of 0.5 McFarland (1.5 × 10^8 ^cfu mL^-1^). A 50 μL aliquot of suspension was added to well A (final concentration of PRE in well A = 200 mg mL^-1^) through to G. All samples were carried out in triplicate. All plates were incubated at 37°C for 24 hours. After incubation 10 μL of broth from each well was spotted onto nutrient agar and incubated at 37°C for 24 hours. After incubation the plates were examined to determine breakpoints by the presence or absence of growth.

### Minimum inhibition concentration determination of PRE/CuSO_4 _combination

The assay was carried out as described above with the following changes: PRE and CuSO_4 _were prepared as before but using four times concentration of half the determined MIC. Addition of the CuSO_4 _was made to the PRE suspension instead of sterile water.

## Results

### Antimicrobial testing

Antibiotic sensitivity profiles were first determined for the clinical isolates and the MRSA isolates were on average resistant to four of the eight antibiotics tested (Table 1). All of the MSSA isolates and the PVL positive CA-MSSA were resistant only to penicillin.

**Table 1:** Antibiotic resistances profile for the *S. aureus *tested.

**Isolates**	**Source**	**Antibiogram**
Methicillin-resistant *Staphylococcus aureus *1	BW	Fluclox, Pen, CXM
Methicillin-resistant *Staphylococcus aureus *2	WS	Fluclox, Pen, Ery, CXM
Methicillin-resistant *Staphylococcus aureus *3	NS	Fluclox, Pen, Ery, Gent, CXM
Methicillin-resistant *Staphylococcus aureus *4	SPT	Fluclox, Pen, Ery, CXM
Methicillin-resistant *Staphylococcus aureus *5	NS	Fluclox, Pen, Ery, CXM
Methicillin-resistant *Staphylococcus aureus *6	WS	Fluclox, Pen, Ery, Gent, CXM
Methicillin-resistant *Staphylococcus aureus *7	WS	Fluclox, Pen, Ery, CXM
Methicillin-resistant *Staphylococcus aureus *8	WS	Fluclox, Pen, Ery, CXM
Methicillin-resistant *Staphylococcus aureus *9	NS	Fluclox, Pen, Ery, CXM
Methicillin-resistant *Staphylococcus aureus *10	U	Fluclox, Pen, Ery, CXM
Methicillin-sensitive *Staphylococcus aureus *1	WS	Pen
Methicillin-sensitive *Staphylococcus aureus *2	WS	Pen
Methicillin-sensitive *Staphylococcus aureus *3	WS	Pen
Methicillin-sensitive *Staphylococcus aureus *4	WS	Pen
Methicillin-sensitive *Staphylococcus aureus *5	WS	Pen
Methicillin-sensitive *Staphylococcus aureus *6	WS	Pen
Methicillin-sensitive *Staphylococcus aureus *7	WS	Pen
Methicillin-sensitive *Staphylococcus aureus *8	WS	Pen
Methicillin-sensitive *Staphylococcus aureus *9	WS	Pen
Methicillin-sensitive *Staphylococcus aureus *10	WS	Pen
PVL producing CA-MSSA 1	WS	Pen
PVL producing CA-MSSA 2	WS	Pen
PVL producing CA-MSSA 3	WS	Pen
PVL producing CA-MSSA 4	WS	Pen
PVL producing CA-MSSA 5	WS	Pen
PVL producing CA-MSSA 6	WS	Pen
PVL producing CA-MSSA 7	WS	Pen
PVL producing CA-MSSA 8	WS	Pen
PVL producing CA-MSSA 9	WS	Pen
PVL producing CA-MSSA 10	WS	Pen

KEY for source: BW: breast wound; WS: wound swab; NS: nose swab; U: urine; SPT: sputum.

Key for antibiotics: cefotixitin (**Fluclox**), penicillin (**Pen**), erythromycin (**Ery**), gentamicin (**Gent**), cefuroxime (**CXM**),

### Antimicrobial activity of PRE with addition of cupric sulphate

**Figure 1:**
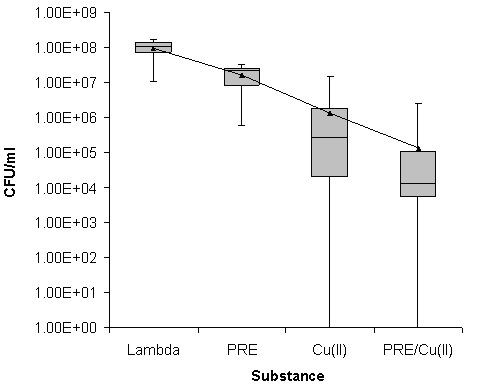
**Box Whisker statistical analysis of the viable count data achieved in relation to the antimicrobial activities of PRE alone and in combination with Cu(II) ions after a 2 hour incubation against 10 clinical isolates of MRSA using Lambda buffer as a control**. (Box represents 25% and 75% quartiles, bar represents median and error bars represent range. Mean cfu mL^-1 ^value shown by black triangle).

For the MRSA isolates, the PRE on its own exhibited no activity (Figure 1). In contrast, the copper (II) ions had some activity of between 10^2 ^to 10^3 ^log cfu mL^-1 ^reduction in growth. However, the PRE/Cu (II) combination exhibited an enhanced activity of 10^4 ^log_10 _cfu mL^-1 ^reduction in growth observed in 50% of the isolates. No detectable level of growth was determined for one isolate; however, a similar result was obtained for the Cu(II) on its own.

**Figure 2:**
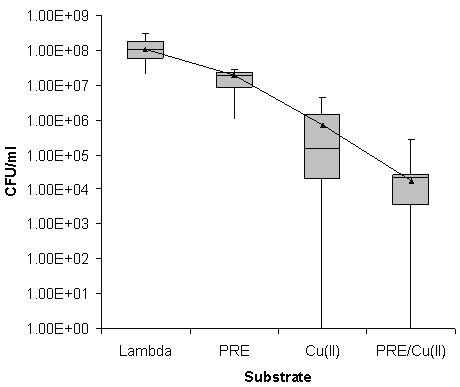
**Box Whisker statistical analysis of the viable count data achieved in relation to the antimicrobial activities of PRE alone and in combination with Cu(II) ions after a 2 hour incubation against 10 clinical isolates of MSSA using Lambda buffer as a control**. (Box represents 25% and 75% quartiles, bar represents median and error bars represent range. Mean cfu mL^-1 ^value shown by a black triangle).

Similar results were observed for the MSSA isolates, with no discernable effect being observed for the PRE alone (Figure 2). However, Cu(II) alone demonstrated a mean reduction of 10^2 ^log_10 _cfu mL^-1^, although four of the isolates showed a reduction of 10^4 ^log_10 _cfu mL^-1^and one isolate recorded no detectable growth. The combination of PRE/Cu(II) showed a mean reduction of 10^4 ^log_10 _cfu mL^-1 ^with only two isolates exhibiting no detectable growth. However, one of these isolates also had no detectable growth with Cu(II) alone.

**Figure 3:**
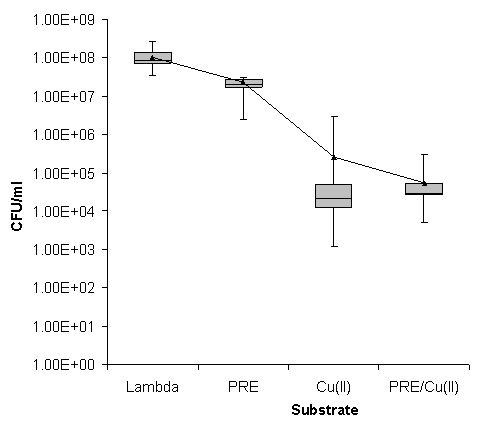
**Box Whisker statistical analysis of the viable count data achieved in relation to the antimicrobial activities of PRE alone and in combination with Cu(II) ions after a 2 hour incubation against 10 clinical isolates of PVL positive CA-MSSA using Lambda buffer as a control**. (Box represents 25% and 75% quartiles, bar represents median and error bars represent range. Mean cfu mL^-1 ^value shown by a black triangle).

For the PVL positive CA-MSSA isolates, once again the PRE alone had no activity against any isolates studied. In contrast to the MSSA and MRSA, the PVL positive CA-MSSA isolates were more sensitive to copper (II) ions which exhibited a moderate reduction in growth of 10^3 ^log cfu mL^-1 ^for 60% of the isolates. Notably, for the remaining 40% of the isolates, less reduction in growth indicated less sensitivity to Cu(II) ions; however, the addition of PRE reduced the growth in these 40% in line with the copper-sensitive 60% (Figure 3).

### Antimicrobial activity of PRE and metal salts with the addition of different concentrations of vitamin C

The combination of Cu (II) sulphate and vitamin C exhibited a reduction in growth of 10^4 ^log_10 _cfu mL^-1 ^compared to the buffer control (data not shown). The addition of vitamin C to the PRE/Cu(II) compound demonstrated similar results to Cu (II) sulphate and vitamin C mixture (data not shown).

### Minimum inhibition concentration determination of PRE and CuSO_4_

Determination of the MIC of PRE and CuSO_4 _individually and in combination are shown in Table 2: PRE had an MIC between 25.0–12.5 mg mL^-1 ^for all isolates tested. The MIC of CuSO_4 _alone ranged from 3.12–0.78 mg mL^-1^, with a mode concentration of 1.56 mg mL^-1^. The combination of PRE: Cu(II) against all isolates of *S. aureus *resulted in values which were half or a quarter of the MIC of PRE or CuSO_4 _alone. Thus, an additive effect was seen against all groups of *S. aureus *for the combination.

**Table 2:** Minimum inhibition concentration of PRE and CuSO_4 _alone and in combination against ten isolates each of MRSA, MSSA and Panton-Valentine Leukocidin positive CA-MSSA.

Isolates type	No. of isolates	MIC (mg/mL)		
		PRE	CuSO_4_	*PRE/CuSO_4_
MRSA	1	12.5	3.125	6.25/1.563
	1	25	1.563	6.25/0.781
	2	25	3.125	12.5/1.563
	3	25	1.563	12.5/0.781
	2	12.5	1.563	6.25/0.781
	1	12.5	1.563	6.25/0.781
MSSA	1	25	1.563	6.25/0.391
	3	25	1.563	12.5/0.781
	1	25	3.125	6.25/0.781
	1	12.5	1.563	3.125/0.391
	1	12.5	0.782	3.125/0.196
	3	12.5	1.563	6.25/0.781
PVL	3	25	1.563	6.25/0.391
	4	25	3.125	6.25/0.781
	2	25	0.781	6.25/0.195
	1	25	1.563	12.5/0.781

*For the PRE/CuSO_4 _combinations the fixed ratio for each component is maintained as shown.

## Discussion

The antimicrobial effects of pomegranate extracts have been well publicised over the last decade [8-10,15,17]. A small number of studies have investigated whether the antimicrobial properties of extracts, such as PRE, can be enhanced with the addition of metal salts [14,15,18]. Stewart and colleagues [14] demonstrated that the combination of PRE and ferrous sulphate could be used a part of a rapid detection for tuberculosis. A recent study by Sivasankaran-Nair and Selwin-Joseyphus demonstrated the antimicrobial effects of vanillin extract (an extract of vanilla) complexes with a number of different metal salts [18]. The antimicrobial activity of these combinations was determined by agar diffusion and results showed the most active complex to be vanillin and copper against the test organisms (*S. aureus, E. coli, K. pneumoniae, P. vulgaris, P. aeruginosa *and *Candida albicans*).

The aim of this study was to extend our previous work to investigate the combination of PRE/CuSO_4 _and vitamin C against a range of clinical isolates of *S. aureus*. Initially the assay was performed as in our previous study with an incubation time of 30 minutes [15]; however, little to no antimicrobial activity was seen against the MSSA isolates. Therefore the incubation of the bacteria with the PRE/CuSO_4 _combination was extended and an optimum time of two hours was determined (data not shown). Against all isolates of *S. aureus *the PRE alone gave results similar to the results of the blank sample (Figures 1, 2 and 3), demonstrating that over the two hour time period the PRE did not affect the bacteria. Although, in this study, the PRE alone did not appear to affect the bacteria, a previous study has demonstrated that PRE does show an antimicrobial activity against a range of bacteria including MRSA [12]. Challenging different types of MRSA with cupric sulphate produced a reduction in cfu mL^-1 ^of between 2 to 3 log_10 _(Figures 1, 2 and 3). The combination of PRE/Cu(II) resulted in a further reduction of a log_10_, compared to the Cu(II) on its own (Figures 1, 2 and 3). The addition of vitamin C to the PRE/CuSO_4 _combination did not improve the antimicrobial properties of the combination against any of the *S. aureus *isolates tested in line with previous observations.

The individual MIC values of PRE and Cu(II) were determined for each isolate and the MIC of PRE on its own was found to vary between 12.5 to 25 mg mL^-1^. These results are similar to those reported by Prashanth *et al*. [17] who recorded MIC values of 25 mg mL^-1 ^against *S. aureus*. However, other studies have reported lower MIC values ranging from 0.5–2 mg mL^-1 ^[7,19] and up to up to 250 mg mL^-1 ^against *S. aureus *[11]. These differences could be due to the extraction method, freshness of the fruit, season and region of its growth. The MIC for CuSO_4 _from this study varied between 0.78–3.12 mg mL^-1^; Arestrup and Hasman [20] determined the MIC of CuSO_4 _against *S. aureus *to be between 2–12 mM. These differences in the MIC values for CuSO_4 _may be due to the different strains of *S. aureus *used. When the MIC combination of PRE and CuSO_4 _was determined it was found that the MIC of both PRE and CuSO_4 _decreased by a factor of one half (i.e. PRE decreasing from 12.5 mg mL^-1 ^to 6.25 mg mL^-1^).

## Conclusion

In conclusion, PRE in combination with Cu(II) ions exhibits additive antimicrobial effects against three classes of *S. aureus*. For MSSA, MRSA and PVL positive CA-MSSA isolates, moderate antimicrobial activities were exhibited by the mixture. The results of this study suggest that further investigations into the active ingredients, the mode of action and potential synergism with other antimicrobials are warranted. This is the first report of the efficacy of pomegranate against PVL positive CA-MSSA isolates.

## Competing interests

The study was in part funded by Nature Therapeutics Ltd. The authors declare that they have no other competing interests.

## Authors' contributions

SWJG, MDF, AFK, DPN participated in the design of the study, analysed the data and wrote the paper. All authors read and approved the final manuscript.

## Pre-publication history

The pre-publication history for this paper can be accessed here:


